# The importance of gaining the public's confidence to use administrative data for public benefit in social science research

**DOI:** 10.23889/ijpds.v1i1.344

**Published:** 2017-04-19

**Authors:** Ilse Verwulgen, Judith Knight

**Affiliations:** 1 Administrative Data Service, University of Essex

## Background

The Administrative Data Research Network (ADRN) is a UK-wide initiative, funded by the Economic and Social Research Council (ESRC). The Network facilitates safe access to linked de-identified administrative data for research which is aimed at providing a benefit to our society. Administrative data research can provide wide ranging and longitudinal evidence for policy makers which therefore has the potential to improve our society.

## Method

Recognising the importance of public confidence and trust to the success of the ADRN, the ESRC commissioned a public consultation to gauge understanding of social research and the reactions to the use of administrative data in research. A comprehensive UK-wide communications and public engagement strategy has been developed. From this a number of initiatives been introduced over the past two years to address public concerns and these have been reviewed, revised and extended as the Network has evolved.

Now to extend the Network's reach, the Administrative Data Research Network is developing a UK National Citizens Panel (CP). The panel will provide a representation of public views on potential changes to Network policy, procedures, governance and service provision issues. The CP will also assist with testing our public facing communications such as events, website and promotional materials.

## Conclusion

This paper presents the previous and current public engagement initiatives that the Administrative Data Research Network has incorporated within its policies and service that enables better knowledge for a better society

Funded by the Economic & Social Research Council, the ADRN, set up as part of the UK Government's Big Data initiative, is a UK-wide partnership between universities, government bodies, national statistics authorities and the wider research community. www.adrn.ac.uk

